# Unraveling the Mystery: A Case Report of Erythrodermic Psoriasis as an Adverse Reaction to Wellbutrin

**DOI:** 10.7759/cureus.88542

**Published:** 2025-07-22

**Authors:** Vincent Blandino, Veera Bommu, Stephen Mbah, Ali J Rizvi

**Affiliations:** 1 Internal Medicine, RWJBarnabas Health Community Medical Center, Toms River, USA

**Keywords:** bupropion, cyclosporine, desquamating, erythrodermic, psoriasis

## Abstract

Understanding unintentional and adverse reactions to medications is key in pharmacologic therapy. These adverse effects can range from mild, well-tolerated symptoms to detrimental consequences for the patient. These symptoms can also range in terms of organ system involvement, some of which may be isolated to only the skin, while others can create end-organ damage and involve multiple systems. Psoriasis, a common inflammatory skin condition, can also be found as an adverse reaction to medications, particularly bupropion, also known as Wellbutrin. We present a rare finding of biopsy-proven erythrodermic psoriasis (EP) in a 40-year-old female with no previous history of psoriasis that occurred shortly after starting bupropion. This report highlights the importance of considering drug-induced adverse reactions in patients presenting with atypical dermatologic manifestations.

## Introduction

Approximately 2% of the US population is impacted by psoriasis, a common inflammatory skin condition affecting millions worldwide [1,2]. It is classified into several subtypes that vary in clinical presentation, such as psoriasis vulgaris, pustular psoriasis, arthropathic psoriasis, and erythrodermic psoriasis (EP) [3]. EP has an incidence of 1-2.25% among individuals with psoriasis, and is the most common cause of erythroderma, encompassing around 25% of all cases, while also being the most serious type of psoriasis [1,2,3]. The condition usually presents as a generalized erythematous, pruritic, and scaling rash that covers at least 90% of the body surface area and has the potential for palmar-plantar or diffuse skin desquamation [1,4]. It is often triggered by environmental factors such as skin trauma, alcohol abuse, and emotional stress; systemic illnesses, including HIV, leukemias/lymphoma, and gout; and pharmaceuticals, such as lithium, cotrimoxazole, and antimalarials [2]. EP also has several complications, including impairment in barrier function of the skin and disturbances in basal metabolic rate, which can lead to massive fluid losses and electrolyte abnormalities [1,2]. Increased cutaneous circulation in EP can also negatively affect patients, leading to high-output heart failure [1,2]. Retrospective studies have established an identifiable trigger for EP in about 53% of cases, and disease recurrence in 15% [2]. Bupropion, a well-known antidepressant, has been associated with several cases of both new onset and exacerbation of psoriasis, especially EP [5,6,7]. We present a case of a 40-year-old female with no history of autoimmune disease or psoriasis who developed EP shortly after starting bupropion.

## Case presentation

A 40-year-old female with a past medical history of anxiety, depression, bipolar disorder, hyperlipidemia, and previously treated Lyme disease presented to the emergency department (ED) with a chief complaint of a diffuse skin rash. The rash had started approximately two weeks ago and first appeared on her palms and the dorsum of her hands, which had later spread diffusely. The rash covered the trunk, extremities, creases, palms, soles, and genitals, but spared the face. She noted that it had started as “mild blotches,” which had since coalesced and were mildly pruritic.

She had visited her outpatient dermatologist after having the rash for three days, and she had been prescribed permethrin due to a suspicion of scabies. Later, it was noted she had been prescribed methylprednisolone and triamcinolone cream; however, we did not have any documentation regarding the reasoning behind these prescribed medications. She visited the ED on day four of the rash, was administered a dose of IV dexamethasone, and was given discharge instructions to follow up with her dermatologist in one week. Over the next three days, her rash worsened despite proper usage of the newly prescribed medications. She returned to the dermatologist on day seven of the rash, where a skin biopsy was performed, and clobetasol was added to her regimen. 

**Figure 1:**
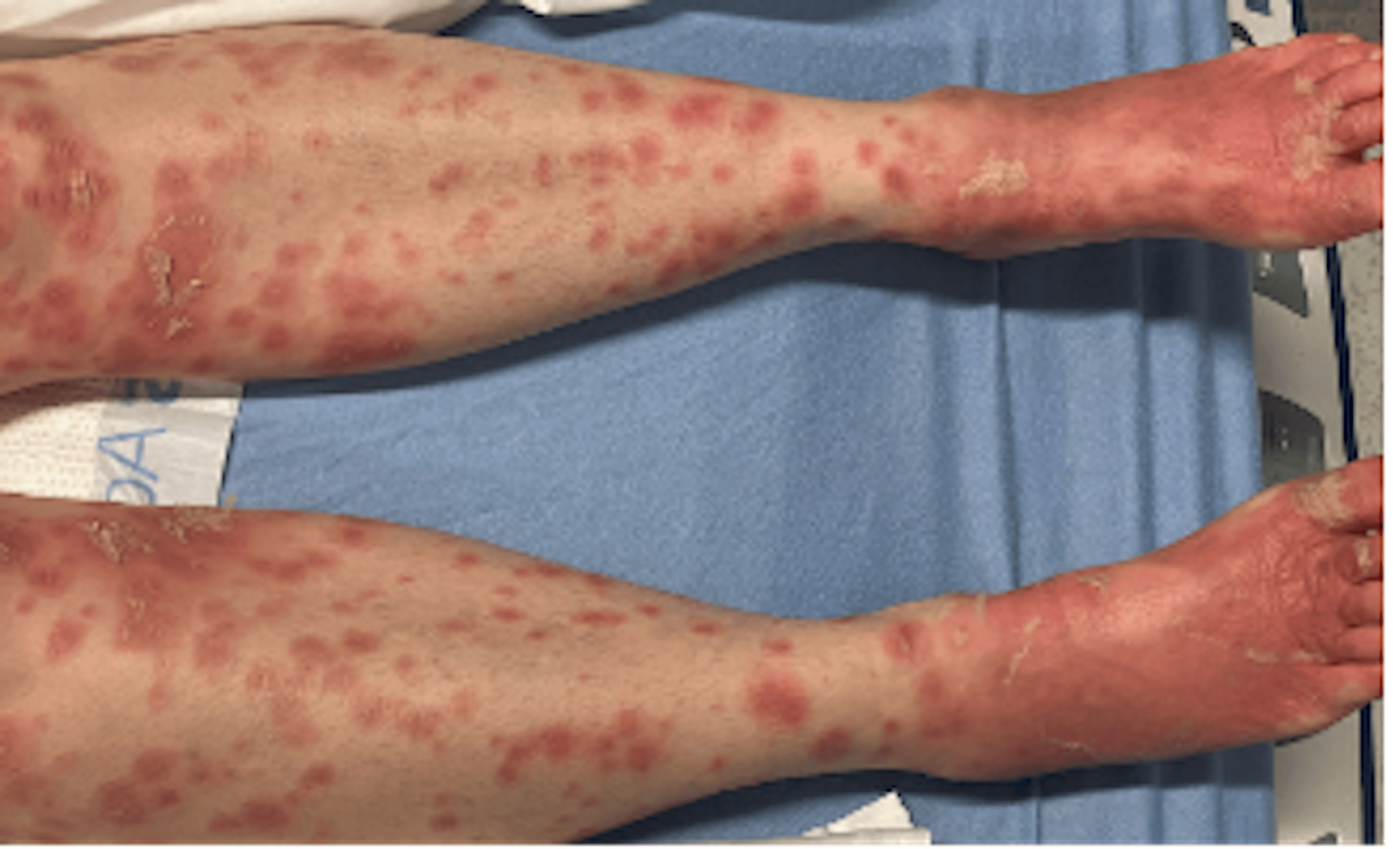
Multiple erythematous macular rash over bilateral lower extremities

**Figure 2:**
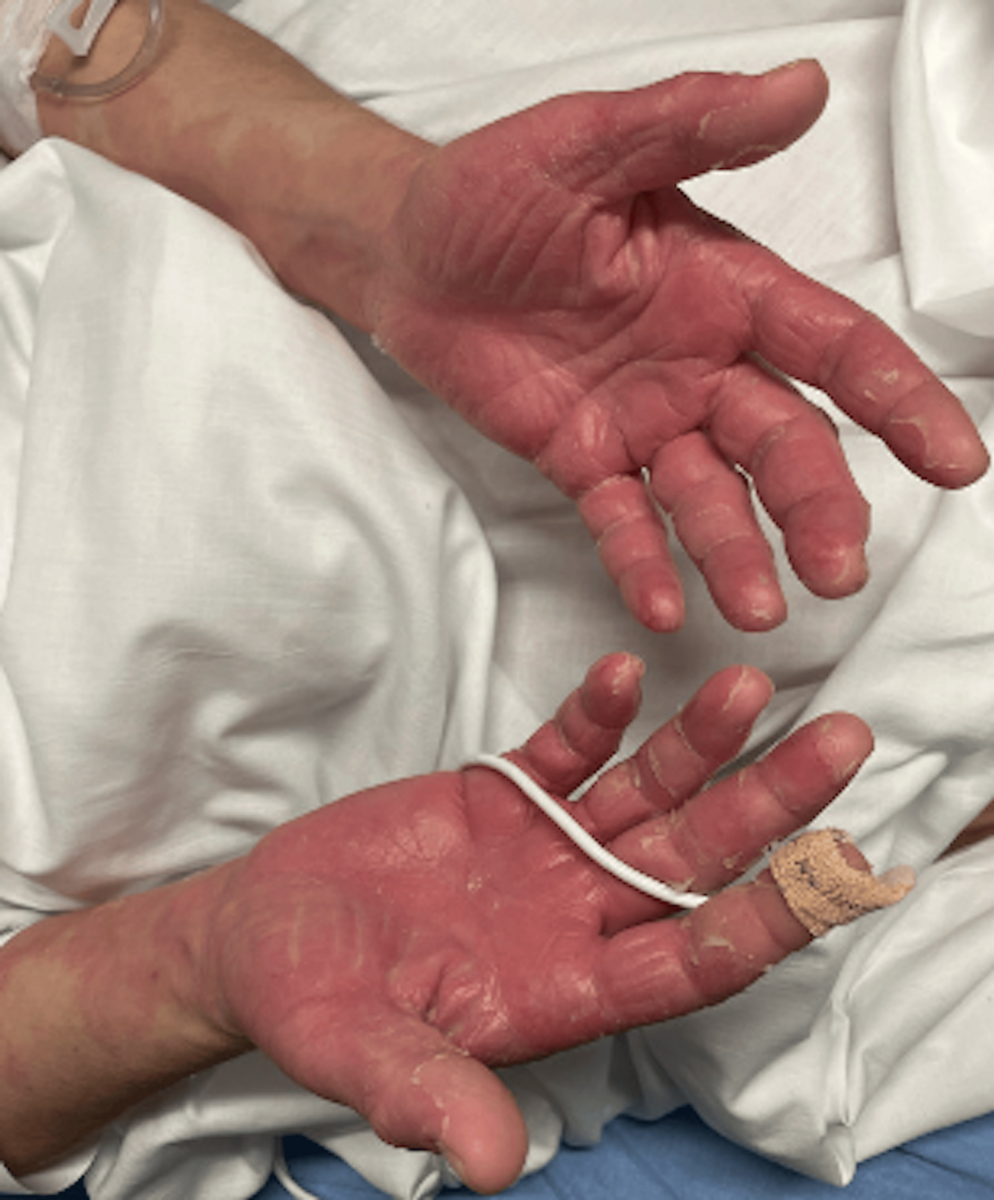
Multiple erythematous macular rash over bilateral palms

**Figure 3:**
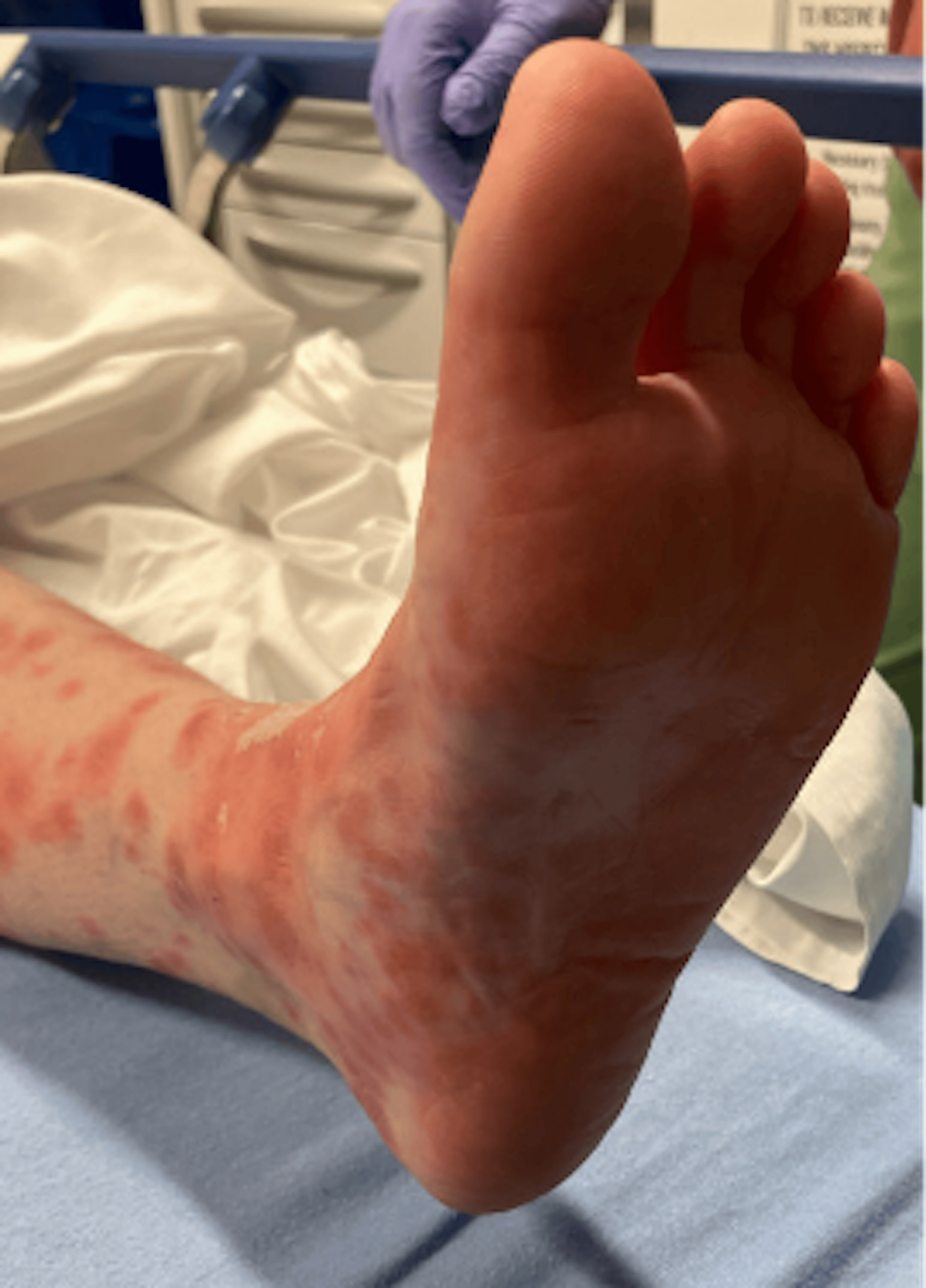
Multiple erythematous macular rash over the sole

She returned to the ED on day 14 of the appearance of rash with complaints of a now sloughing, flaking, diffuse rash, as well as malaise. She denied any recent travel, sick contacts, noticeable bug bites, changes in soaps/detergents, or history of similar rashes. Of note, she had recently started two new medications two weeks before the onset of the rash: Wellbutrin (bupropion) and trazodone. She had stopped taking these two new medications four days after the rash began. She complained of mild, diffuse pruritus and lip swelling. The remainder of the review of systems (ROS) was negative. She was found to be in moderate distress, afebrile, with a heart rate (HR) of 90, respiratory rate (RR) of 19, blood pressure (BP) of 140/84 mmHg, and saturating at 97% on room air. On physical exam, she had multiple erythematous macular lesions (Figures 1, 2, 3) noted covering approximately 75% BSA, sparing the eyelids and mucous membranes. The macules were blanchable and in various sizes, with peeling lesions noted, particularly in the lower extremities and palms. She also had diffusely erythematous and slightly warm-to-touch hands and feet bilaterally, with erythema extending to wrists and ankles. Nikolsky's sign was negative.

The patient was initially treated with broad-spectrum intravenous (IV) antibiotics and steroids, in addition to a trial of IV cyclosporine 5 mg/kg divided three times a day. The differentials at that time were generalized bullous fixed drug eruptions secondary to trazodone vs. pityriasis rosea-like drug eruptions due to Wellbutrin vs. Stevens-Johnson syndrome (SJS) being less likely. With the administration of these medications, the rash improved clinically, and the patient noted decreased pruritus and generalized improvement as well. Her pending skin biopsy was finally conducted on day 16 of the appearance of the rash, revealing EP. She was subsequently discharged home on oral cyclosporine 100 mg three times daily in a stable condition with a dermatologist follow-up appointment.

## Discussion

Bupropion is a modulator for dopaminergic and noradrenergic signaling, commonly prescribed for depression, smoking cessation, and various other off-label treatments [6]. It is also generally well tolerated, with common side effects including dry mouth, constipation, headache, nausea, agitation, insomnia, and weight loss [6]. Although rare, angioedema and serum-sickness-like reactions have also been documented reactions associated with bupropion [4]. Several case reports have noted more severe effects of bupropion-associated psoriasis and cases of EP requiring hospitalization [4,5,7]. Furthermore, in the absence of other triggering factors, such as infection or emotional stress, the possibility of drug-related causes must be considered [5]. Our patient did not report any triggering factors, leading us to conclude that bupropion was likely the offending agent. It is worth mentioning that a flare can persist even if the suspected offending agent is discontinued [5]. This was seen in our case, where the patient's rash persisted despite discontinuing the medication. 

The pathogenesis of psoriasis involves T cell activation, which produces lymphokines that stimulate skin proliferation, causing keratinocytes to release cytokines that further strengthen T cells [3]. Th1 cells, a division of CD4+ T lymphocytes, secrete cytokines interferon (IFN)-γ, interleukin (IL)-2, and tumor necrosis factor beta (TNF-β), while Th2 cells secrete IL-4, -5, -6, and -10 [3]. Studies assessing the pathogenesis of psoriasis subtypes have shown that EP involves significantly higher levels of IL-4 and IL-10, a dramatically lower ratio of Th1/Th2 levels, and a statistically significant difference in T-box expressed in T-cells/GATA-binding protein-3 (GATA-3), as well as IFN-γ/IL-4, when compared to psoriasis vulgaris [3]. In essence, the imbalance in these two cell lines is likely a main driver for EP, where Th1 induces disease, and Th2 promotes inflammation, and the combination of these factors proves advantageous towards the severity of the disease [3].

The mechanisms of drug-induced psoriasis are not yet entirely understood [5,7]. However, some well-described medications, such as imiquimod, can fit well into the pathophysiology of psoriasis itself [5]. Imiquimod, a toll-like receptor 7 agonist, can activate dendritic cells to produce interferons, which themselves have a key role in psoriasis as previously described [5]. Ultimately, the exact mechanism of how bupropion can trigger EP is unknown, but we hypothesize that there could be similar underlying mechanisms unbeknownst to the medical community currently [7].

Our patient was treated with cyclosporine, leading to significant symptom reduction. A potent immunosuppressive agent that blocks IL-2 transcription to prevent the growth of T cells, it is considered a critical first-line drug for unstable cases of EP [2]. Data from other reports advise cyclosporine monotherapy at 1.5-4.2 mg/kg/day for two weeks to four months, as it has led to complete remission in most patients, and a high response rate in general at 94% [2]. Since nephrotoxicity is the most serious possible adverse effect of cyclosporine, a rise in serum creatinine of more than 30% necessitates lowering the dosage or stopping the medication altogether [2].

## Conclusions

This report underscores the importance of considering drug-induced adverse reactions in patients presenting with atypical dermatologic manifestations. Clinicians should be vigilant for potential triggers, such as medications, and promptly discontinue them if suspected, especially in the absence of more common triggers. Further research is warranted to elucidate the true pathogenesis and optimal management strategies for drug-induced EP.
